# Supplementary material for: Coin Tossing Explains the Activity of Opposing Microtubule Motors on Phagosomes
Source: Curr Biol. 2018 May 7;28(9):1460–1466.e4. doi: 10.1016/j.cub.2018.03.041 (PMC5954897; doi:10.1016/j.cub.2018.03.041)
Supplement: Document S1. Figures S1–S4 [file mmc1.pdf]

Current Biology, Volume *28*

Supplemental Information

Coin Tossing Explains the Activity of Opposing Microtubule Motors on Phagosomes

Paulomi Sanghavi, Ashwin D'Souza, Ashim Rai, Arpan Rai, Ranjith Padinhatheeri, and Roop Mallik

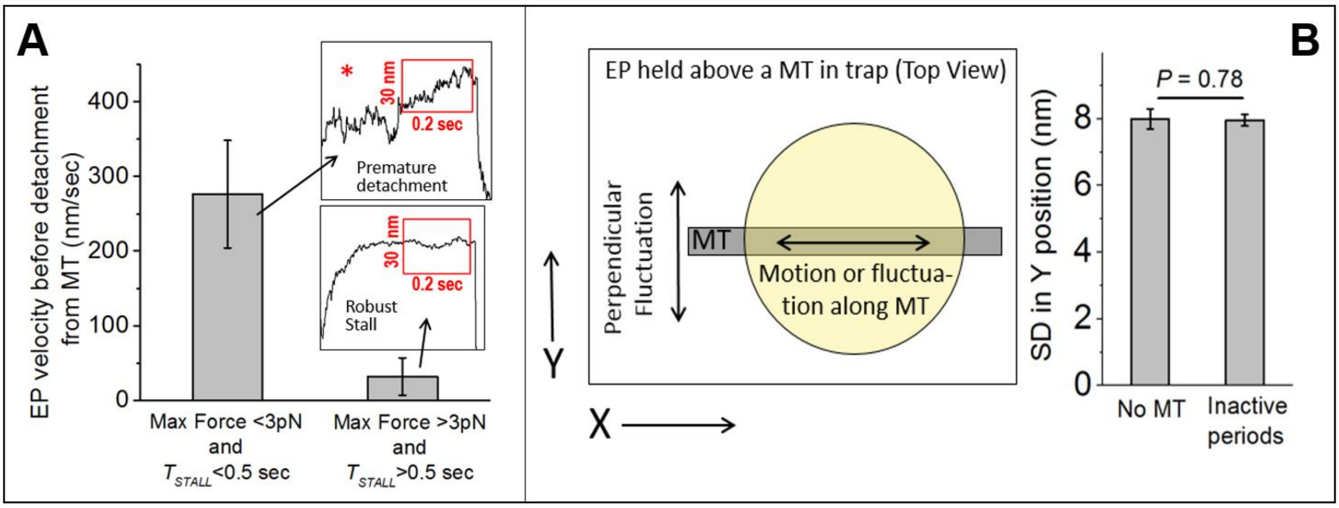

Figure S1. Scoring stalls in the trap and absence of motor activity during inactive periods, related to Figure 1 and Figure 2

A) Identification of premature detachment of Kinesin in an optical trap. Plus directed stalls were divided into two categories based on the criterion mentioned on X-axis of this Figure. The velocity in a 200ms window prior to detachment was measured by linear fitting. The insets show examples of each category and the boxed regions used to calculate velocity. Significantly higher velocity was seen in the first category (red star), suggesting that these are premature stalls.

B) Left Panel:- The SD in position (i.e. the fluctuation) of EPs along Y direction in an optical trap, shown as a schematic. The SD along Y (and not X) was chosen to minimize effect of motor-driven excursions along X.

Right panel:- **"No MT"**-- EPs were caught in the optical trap and held above the coverslip in absence of MTs. **"Inactive periods"** -- examples are shown as yellow boxes in Figure 1A. EPs resided at the centre of the optical trap and did not show significant motion along the MT (i.e. in X direction) during Inactive periods. The SD in position along Y direction during "Inactive period" is plotted. This is equal to the SD for EPs that had no MT underneath them. This suggests that EP-associated motors do not attach to the MT during inactive periods.

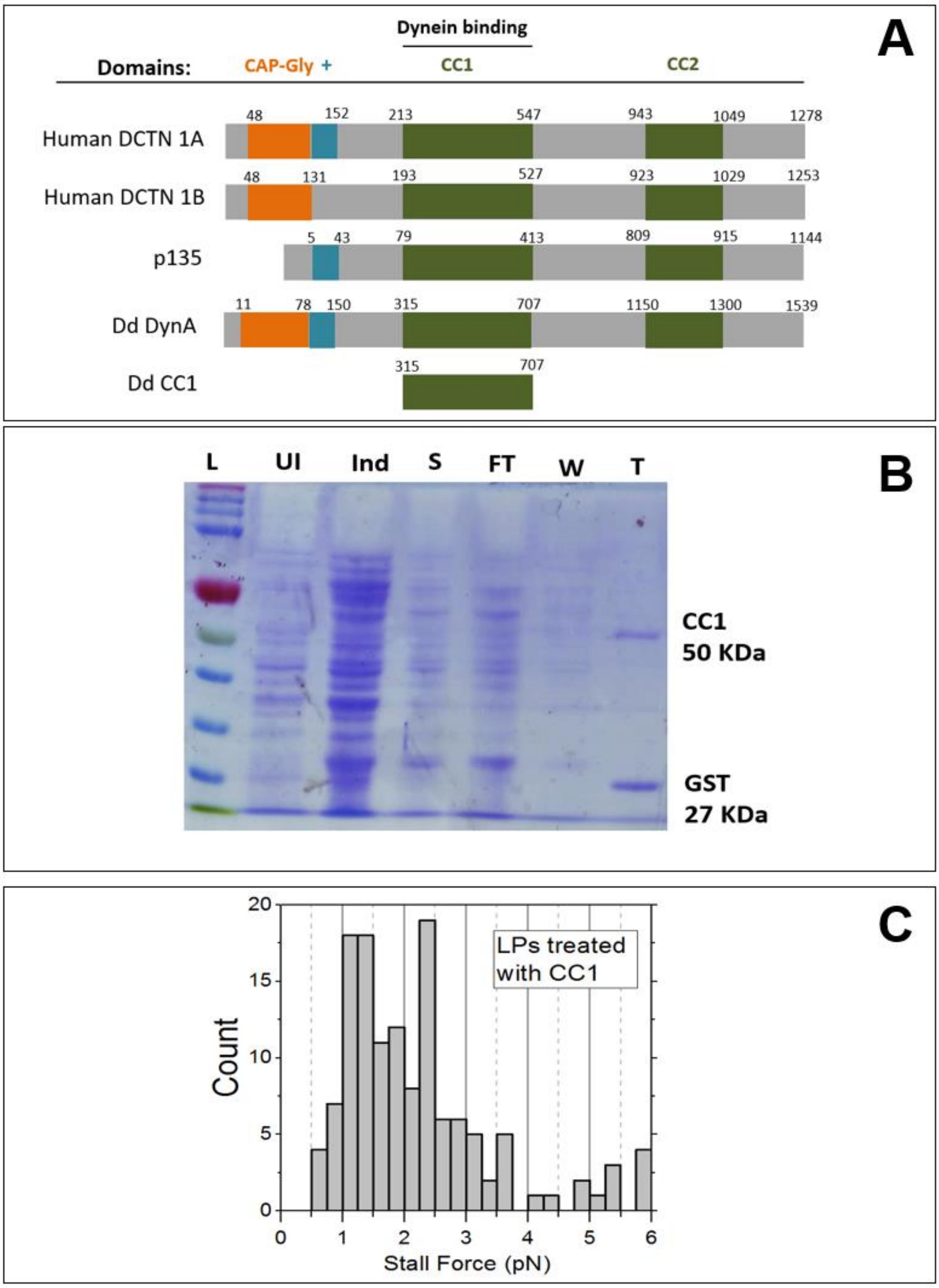

Figure S2. CC1 domains, purification and force measurement of LPs, related to Figure 3

A) Schematic representations of dynactin/p150 isoforms from Human and *Dictyostelium discoideum* (Dd). The CAP–Gly, basic (+), coiled-coil 1 and 2 (CC1 and CC2) domains for each isoform are labeled. The cloned fragment used in motility inhibition assays (Dd CC1) is shown. Numbers indicated on each isoform refer to their corresponding amino-acids.

B) GST-CC1 was purified from BL21 pLys cells using GST sepharose beads. Following are the fractions loaded on the gel. L: Protein Ladder, UI: Uninduced sample, Ind: Induced sample (1mM IPTG, 25 degrees C, 5 hrs), S: Soluble fraction post lysis and spin FT: Flow through W: Wash T : Fraction collected after thrombin cleavage.

C) Stall force histogram for LPs treated with 1 micromolar CC1. The bin size is 0.25pN. Very few counts are seen above 4pN force. The lowest peak is seen at ~1.2pN, suggesting that this is the force exerted by single dynein on CC1 treated LPs. The peak at ~2.2pN is suggestive of force from a pair of dynein motors (see main text).

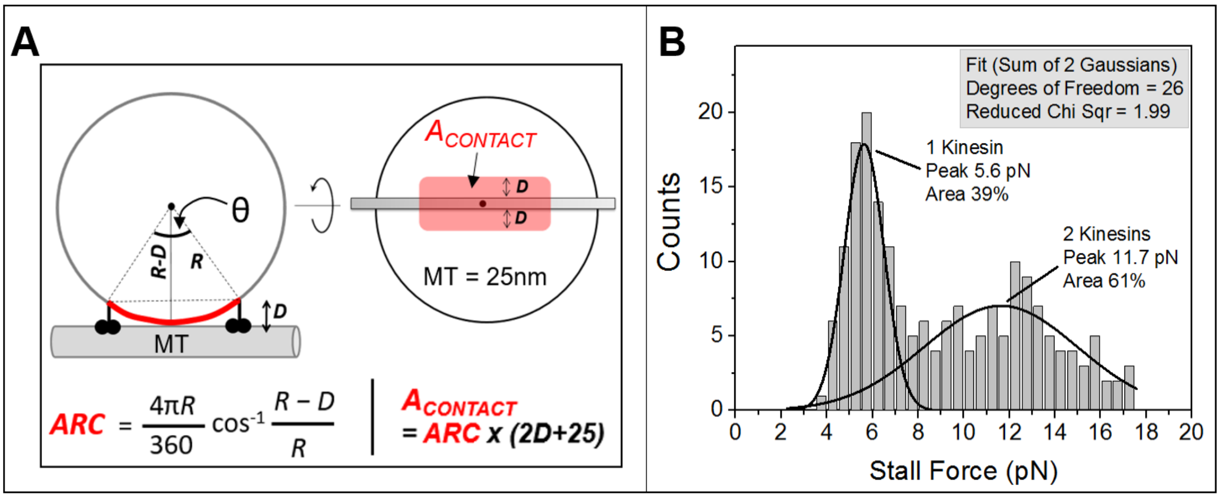

Figure S3. Geometry of phagosome-MT contact and Kinesin stall force histogram, related to Figure 1 and Figure 4

A) Left panel: A spherical cargo of radius R contacts the MT with motors of length *D*. The red line represents an arc from where motors can bind the MT. Motors associated with the cargo outside this region are not long enough to bind the MT. The length of this arc is shown, and the angle subtended by it at the cargo centre (θ) is also shown. **Right panel:** The cargo is rotated as shown. Motors present along the arc on either side of the MT can engage if the perpendicular distance between the point of attachment on the cargo and the MT is less than the length of the motor. The area $A_{CONTACT}$ on the cargo from where motors can contact the MT is shown in red. This area is a rectangle with length equal to the length of the arc and breadth equal to *D+25+D*. For 750nm dia cargoes, $A_{CONTACT}$ is ~4% of the total surface area.

B) Histogram of stall forces of kinesin measured on EPs purified from *Dictyostelium*. The distribution can be fitted to two individual Gaussians: one with a peak at 5.6pN corresponding to 1 kinesin events and other with a 2 kinesin peak at 11.7pN. The gray box reports the fit parameters.

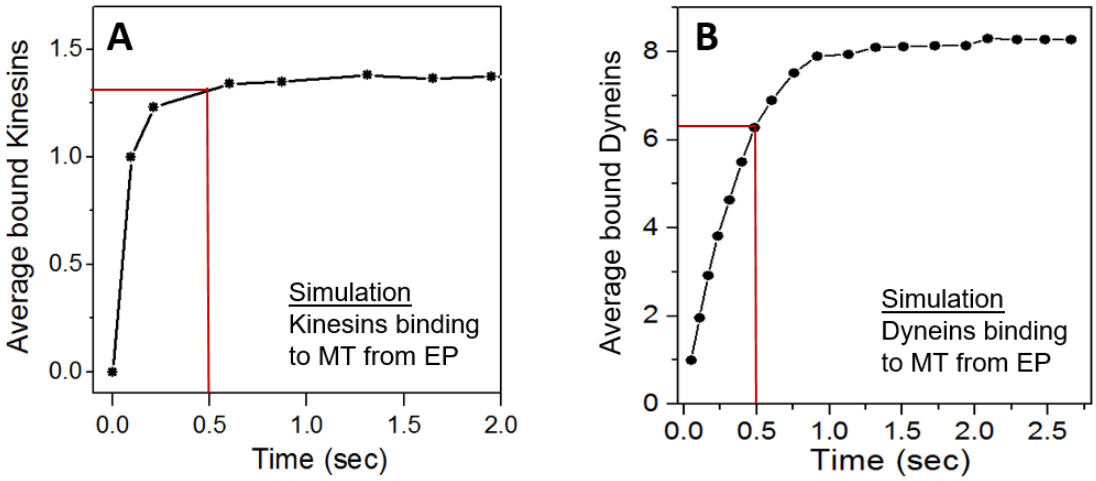

Figure S4, related to Figure 4

Average number of MT-bound motors (simulation).

A) Average number of bound kinesins as a function of time obtained from simulations. 2 kinesins are placed close to a MT (i.e. within $A_{CONTACT}$) and allowed to bind/unbind from the MT (see main text for more details).

B) Average number of dyneins bound to MT as a function of time obtained from simulations. 10 dyneins are placed close to a MT (i.e. within $A_{CONTACT}$) and allowed to bind/unbind from the MT (see main text for more details).
