# Supplementary material for: Coin Tossing Explains the Activity of Opposing Microtubule Motors on Phagosomes
Source: Curr Biol. 2018 May 7;28(9):1460–1466.e4. doi: 10.1016/j.cub.2018.03.041 (PMC5954897; doi:10.1016/j.cub.2018.03.041)
Supplement: Document S2. Article plus Supplemental Information [file mmc2.pdf]

Report

Current Biology

Coin Tossing Explains the Activity of Opposing Microtubule Motors on Phagosomes

Highlights

- Optical trapping data on phagosomes analyzed mathematically
- Dynein and kinesin function stochastically and independently of each other
- Choice between these motors can be explained by tossing of a hypothetical coin
- Regulatory factors may generate polarized transport by biasing this coin

Authors

Paulomi Sanghavi, Ashwin D'Souza, Ashim Rai, Arpan Rai, Ranjith Padinhatheeri, Roop Mallik

Correspondence

roop@tifr.res.in

In Brief

Sanghavi et al. analyze optical trapping data on phagosomes to propose that sub-cellular localization of organelles inside cells can be understood in terms of a hypothetical coin toss that decides motor protein activity (dynein versus kinesin) on the phagosome.

Sanghavi et al., 2018, Current Biology *28*, 1460–1466
May 7, 2018 © 2018 The Author(s). Published by Elsevier Ltd.
https://doi.org/10.1016/j.cub.2018.03.041

CellPress

Current Biology
Report

Coin Tossing Explains the Activity of Opposing Microtubule Motors on Phagosomes

Paulomi Sanghavi,[1,3] Ashwin D'Souza,[1,3] Ashim Rai,[1] Arpan Rai,[1] Ranjith Padinhatheeri,[2] and Roop Mallik[1,4,*]
[1]Department of Biological Sciences, Tata Institute of Fundamental Research, Mumbai 400005, India
[2]Biosciences and Bioengineering, Indian Institute of Technology Bombay, Mumbai 400076, India
[3]These authors contributed equally
[4]Lead Contact
*Correspondence: roop@tifr.res.in
https://doi.org/10.1016/j.cub.2018.03.041

SUMMARY

How the opposing activity of kinesin and dynein motors generates polarized distribution of organelles inside cells is poorly understood and hotly debated [1, 2]. Possible explanations include stochastic mechanical competition [3, 4], coordinated regulation by motor-associated proteins [5–7], mechanical activation of motors [8], and lipid-induced organization [9]. Here, we address this question by using phagocytosed latex beads to generate early phagosomes (EPs) that move bidirectionally along microtubules (MTs) in an *in vitro* assay [9]. Dynein/kinesin activity on individual EPs is recorded as real-time force generation of the motors against an optical trap. Activity of one class of motors frequently coincides with, or is rapidly followed by opposite motors. This leads to frequent and rapid reversals of EPs in the trap. Remarkably, the choice between dynein and kinesin can be explained by the tossing of a coin. Opposing motors therefore appear to function stochastically and independently of each other, as also confirmed by observing no effect on kinesin function when dynein is inhibited on the EPs. A simple binomial probability calculation based on the geometry of EP-microtubule contact explains the observed activity of dynein and kinesin on phagosomes. This understanding of intracellular transport in terms of a hypothetical coin, if it holds true for other cargoes, provides a conceptual framework to explain the polarized localization of organelles inside cells.

RESULTS

Bidirectional Force Generation by Motors on Phagosomes in a Reconstituted Assay

Dictyostelium cells were allowed to ingest beads for a short duration, followed by cell lysis and centrifugation to purify buoyant early phagosomes (EPs) [9, 10]. Individual EPs were caught in an optical trap and held above a single polarity-labeled microtubules (MT) [11]. Endosomes in *Dictyostelium* are driven by the Unc104 kinesin [3, 12, 13], which is likely the plus-directed motor on EPs. Figure 1A shows force generation by kinesin and dynein on two representative EPs. The force from motors (F_M) during an excursion (X) against the trap is calculated from the trap stiffness (K_T) using $F_M = K_T \times X$ (see scale bar, Figure 1A). Accurate determination of K_T is possible by standard techniques [10]. A histogram of plus and minus forces from motors on EPs was presented earlier by us [9], with the maximum force in both directions being ~15 pN. Plus-directed motors showed two peaks at ~6 and ~12 pN, but minus directed motors showed peaks with smaller (~2 pN) periodicity and a major peak at ~6 pN [9]. Because a single *Dictyostelium* dynein generates ~1.1 pN force [3], we have speculated that dynein is recruited in pairs to cellular cargos [9]. Since *Dictyostelium* kinesin generates ~6 pN force [3], this situation corresponds to 1 or 2 strong kinesins in force-balanced competition against a large number of weak dyneins (~6 or more; see further discussion later). This may permit regulation of bidirectional transport [3, 14].

Activation of Dynein and Kinesin Can Be Understood as Coin Tossing

We counted an almost equal fraction of kinesin (*K*)- and dynein (*D*)-driven stalls (47% versus 53%; 90 EPs used). *DK* and *KD* event pairs contain reversals, but *KK* and *DD* do not (Figure 1A). Some low-force events (red stars) were not counted as stalls (see Figure S1A). Figure 1B shows the fractional occurrence of all event pairs on EPs. Interestingly, each fraction is ~25% of the total. Computer-simulated tossing of a fair coin to generate heads (*H*) and tails (*T*) shows that the occurrence of HH/TT/HT/TH event pairs (Figure 1C) matches very closely with the 25% occurrence of *DK*/*DD*/*KD*/*KK* stalls. Therefore, activity of any class of motors is equally likely to be followed by the same or opposite motors (a fair coin). This process appears to have no memory, as in a Markov chain where motor classes are activated stochastically and with equal probability to facilitate efficient sampling of cellular space via back-and-forth motion of the EPs [15–17]. Note multiple $KD \rightarrow DK \rightarrow KD \rightarrow DK \rightarrow KD$ reversals of the first EP in Figure 1A. We have reported optical trapping of motile phagosomes inside mouse macrophage cells [18]. Similar to the *in vitro* experiments, phagosomes reverse direction (Figure 1D) and also escape from the trap (Figure 1E) inside cells. The ability to reverse direction against opposing force may be important for vesicle transport in the crowded cytoplasm [1, 19].

Rapid Reversals of EPs

Inactive periods within an event pair (yellow boxes in Figure 1A) are where the EP stays at the trap center, and no motors engage

 © 2018 The Author(s). Published by Elsevier Ltd.
This is an open access article under the CC BY license (http://creativecommons.org/licenses/by/4.0/).

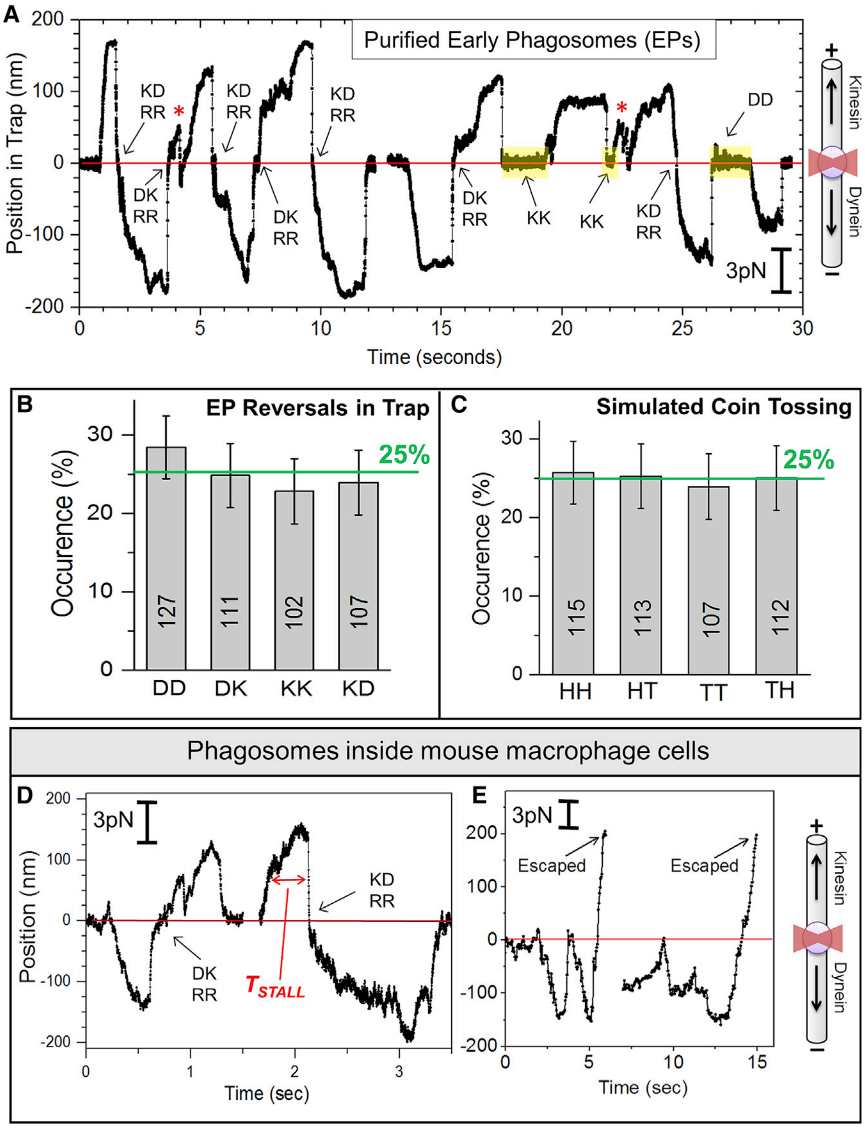

Figure 1. Reversals of Purified Phagosomes and Phagosomes inside Cells in an Optical Trap

(A) Position-time traces of two EPs in an optical trap. A schematic (not to scale) depicts orientation of MT with an EP (sphere) in the trap (red). Transitions between dynein and kinesin are indicated. For example, *KD* depicts a kinesin stall followed by a dynein stall. Inactive periods (yellow boxes) are when the EP sits at the trap center. Reversals with an intervening inactive time of <0.5 s are labeled as RRs (rapid reversals, see text). All reversals in this particular figure are RRs.

(B) Bar graph depicting the fractional occurrence of different types of event pairs *DD*, *DK*, *KK*, and *KD*. A total of 447 event pairs were analyzed. Actual number of events-pairs observed for each type is indicated. The 25% level is indicated. Error bar, SEM.

(C) Fractional occurrence of head (*H*) and tail (*T*) event pairs in computer-simulated tossing of a fair coin.

(D) Stalls of latex bead phagosomes inside a J774.2 cell (mouse macrophage). RR events for *DK* and *KD* event pairs are marked. T_{STALL} is the width of a stall force record at half-maximal force.

(E) Stalls on latex bead phagosomes inside a J774.2 cell, followed by reversals and escape from the trap.

See also Figures S1 and S3.

the MT (also see Figure S1B). Reversals without an intervening inactive period will be called rapid reversals (RRs; see Figure 1A). Note that data are acquired using a quadrant detector at high (2 KHz) sampling rate. The observation of RRs suggests that opposing motors must attach to the MT simultaneously or in rapid succession to cause a transient tug-of-war [3]. To check whether tug-of-wars happen on EPs, data on RRs were compared to (only) kinesin-coated beads where no tug-of-war is possible. Figure 2A shows a stall of a kinesin-coated bead. The box encloses a transient "fly-back" region where the cargo (bead or EP) is two SDs away from its fluctuations at the trap center (e.g., fluctuation within yellow boxes in Figure 1A). The fly-back for kinesin-coated bead is completed within a few milliseconds (Figure 2A; adjacent data points are 0.5 ms apart). Similarly, the fly-back for *DD* stalls on EPs is also completed within a few milliseconds (Figure 2A, middle panel). Because both of these stalls have a post-stall inactive period (yellow boxes), motors therefore did not attach to the MT immediately after the fly-back.

Figure 2A (right panel) magnifies the fly-back during reversals for *KD* and *DK*-type RRs on EPs. Here, the fly-back of one EP (labeled *KD*-RR with tug-of-war) is much slower than the kinesin-coated bead or *DD* events. However, the fly-back of another EP (labeled *KD*-RR without tug-of-war) is rapid and similar to the kinesin-coated bead. This difference is also seen between the last two reversals labeled (*DK*-RR with tug-of-war and *DK*-RR no tug-of-war). Figure 2B shows the fly-back time for kinesin-beads, *KK*/*DD* and *KD*/*DK*-type RRs. The data for RRs appear to separate into a fast and a slow population. We interpret this slowing to arise from a tug-of-war between opposing motors. Figure 2C shows the fraction of fast (RR-no-ToW) versus slow (RR-with-ToW) fly-backs for *DK* and *KD* reversals. These data support the conjecture that opposing motors on EPs are activated stochastically and may therefore engage occasionally in tug-of-war. The molecular properties of motors (K_{ON} and K_{OFF} to MTs, force, etc.) and the surface density of kinesin and dynein on endosome/phagosome membranes [3, 9] therefore appear optimized to ensure intermittent, but not continuous tug-of-wars.

No Evidence for Mechanical Activation of Opposing Motors

Knockdown of kinesin-1 or dynein abrogates peroxisome motion in both directions [8]. However, replacing kinesin-1 by a different plus-directed motor (or dynein by a minus-directed motor) restores motility to varying degrees. It is suggested that one class

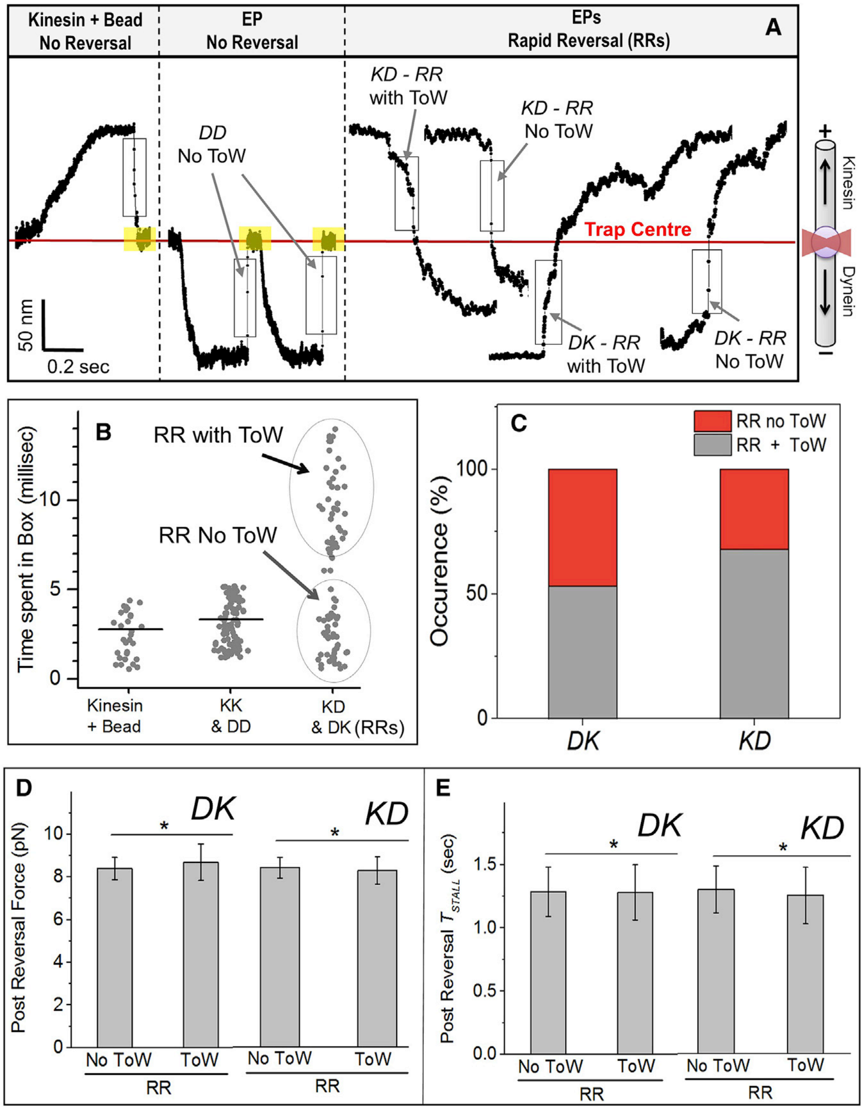

Figure 2. Intermittent Tug-of-War between Motors, but No Mechanical Activation

(A) Representative stalls of a kinesin-coated bead, a *DD* event on an EP (not RR), and *KD* and *DK*-type RRs with tug-of-war (ToW) and without ToW for EPs. Boxes are the "fly-backs" (see main text). Note inactive periods (yellow boxes) after stalls for kinesin-coated bead and *DD*-no-ToW case. No inactive periods are seen for RR events.

(B) Time spent in the fly-back region (see boxes in Figure 2A). Horizontal lines are mean values. Two populations are apparent in the *KD* and *DK*-type RRs (circled). *KK* and *DD* data are pooled. *KD* and *DK* data are pooled.

(C) Frequency of RRs with and without ToW (see main text for basis of this classification).

(D) Post-reversal stall force for RR events. Error bar, SEM.

(E) T_{STALL} for RR events. Error bar, SEM.

See also Figure S1.

of motors mechanically activates the opposing motors [8] when they pull against each other (as in a tug-of-war). If motors get activated on EPs via tug-of-war, then the post-reversal stall force should be higher for "RR with tug-of-war" events as compared to "RR without tug-of-war." However, no such difference was observed (Figure 2D). Pre-reversal stall forces also exhibited no difference. We have earlier used T_{STALL} (time spent above half-maximal force; see Figure 1D) to quantify tenacity of motors [18]. "RR with tug-of-war" and "RR without tug-of-war" events showed similar T_{STALL} (Figure 2E). Therefore, we find no evidence for mechanical activation of motors on phagosomes.

CC1 Domain Removes Dynein from EPs but Has No Effect on Kinesin Function

If indeed opposing motors function stochastically and independently on EPs, inhibiting one motor should have no effect on the other [1, 19]. The dynein-binding CC1 domain of *p150 glued* subunit of *Dictyostelium* dynactin was used to test this possibility (STAR Methods; Figures S2A and S2B). Late phagosomes (LPs) purified from *Dictyostelium* exhibit predominantly dynein-driven motion with rare activity of kinesin [9]. Incubation of LPs with CC1 peptide removed dynein from LPs (Figure 3A), possibly along with other dynein co-factors. Rab7 (LP marker) was not removed by CC1. CC1 induces a conformational change in dynein [20], and such changes may reduce dynein's affinity to the phagosome membrane and/or to accessory proteins. CC1 inhibited LP motion in a dose-dependent manner allowing us to estimate an optimal CC1 concentration (= 1 μM) for inhibition of dynein motility on EPs (Figure 3B). CC1-treated EPs moved only in plus direction (Figure 3C; compare with Figure 1A). CC1 did not change the frequency (Figure 3D), force (Figure 3E), or T_{STALL} (Figure 3F) for kinesin-driven events on EPs. Therefore, abrogation of dynein activity had no apparent effect on kinesin. This suggests that kinesin functions independently of dynein on the EP and is consistent with accumulation of phagosomes at the periphery of CC1-treated cells [18]. It was presumed that inhibiting one motor should increase activity/transport of cargo by the opposing motor [1, 5, 19]. This would be expected if opposing motors engage in continuous tug-of-war. However, motors engage in intermittent (and not continuous) tug-of-war on endosomes [3] and EPs (Figure 2A). Inactivating dynein simply stops these tug-of-wars but has no observable impact on kinesin.

Force Measurements on CC1-Treated LPs

A recent report suggests that single dynein within artificial dynein-dynactin-BicD (DDB) complexes assembled on beads generates ~4.3 pN force [21]. Dynein does not generate such a high force on native-like phagosomes (STAR Methods). We have suggested that two dyneins (each generating ~1.1 pN)

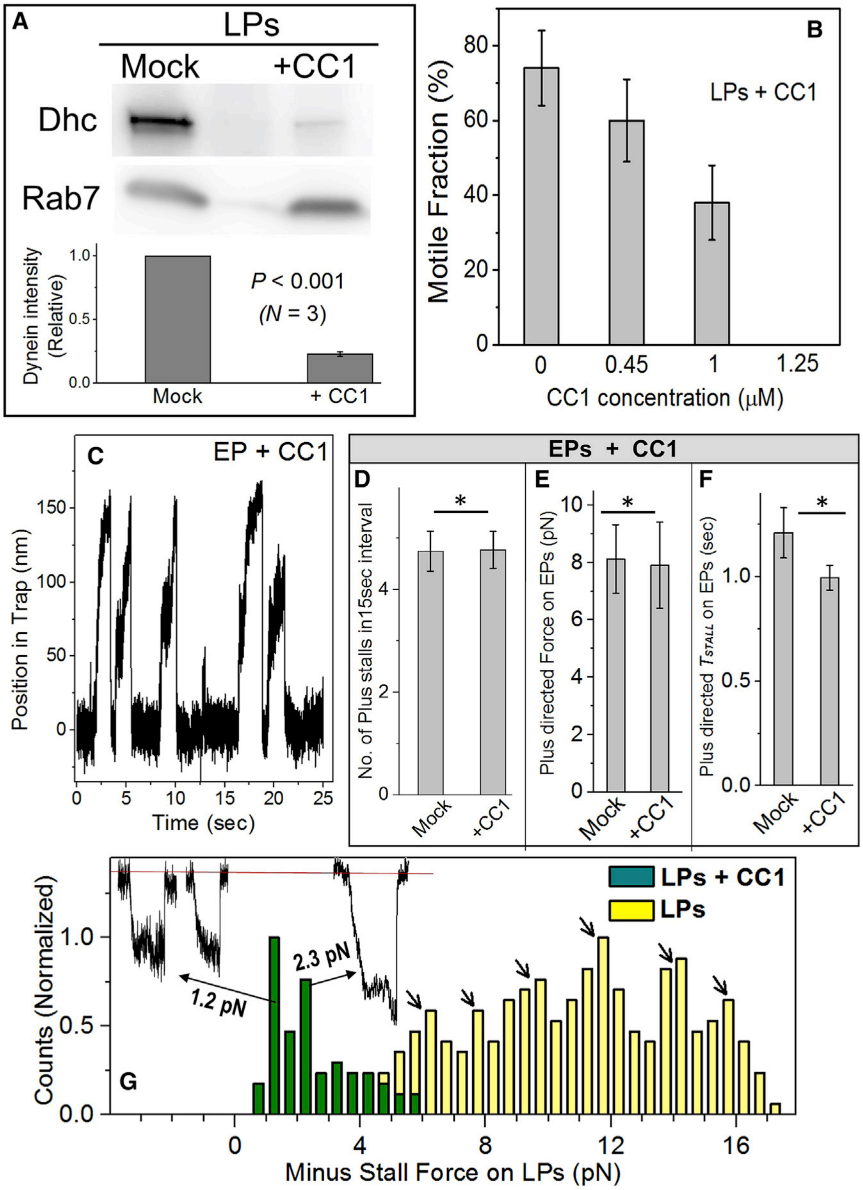

Figure 3. CC1 Treatment-Opposing Motors Function Independently on Phagosomes

(A) LPs were divided into two equal groups. One group was incubated with only buffer (mock) and the other with CC1 in the same buffer. Western blot shows dynein heavy chain (Dhc) and Rab7 levels on mock- and CC1-treated LPs. Lower panel shows quantification across three experiments. Errors bars, SEM.

(B) Motile fraction of late phagosomes (LPs) as a function of CC1 concentration in an *in vitro* motility assay. LPs were held above a MT for 20 s and scored for motion/no motion in the trap. This experiment was repeated across three LP preparations. Error bars, SEM.

(C) Representative stalls of an EP incubated with CC1 at a concentration of 1 μM. Minus-directed stalls are completely abrogated; only plus-directed stalls are seen.

(D) The frequency of plus-directed stalls is same for mock- versus CC1-treated EPs. The number of stalls in a 15-s interval is plotted to represent the frequency. The asterisk indicates no statistical significance (two-tailed t test).

(E) The stall force of plus-directed stalls is same for mock- versus CC1-treated EPs. Mean is indicated by lines. The asterisk indicates no statistical significance.

(F) The T_{STALL} force of plus-directed stalls is same for mock- versus CC1-treated EPs. Mean is indicated by lines. The asterisk indicates no statistical significance.

(G) Histogram of stall force for CC1-treated and untreated LPs. Bins are 0.5 pN wide. For CC1-treated LPs, a low-force population with peaks at ~1.2 and ~2.3 pN is seen, suggesting that single dynein generates ~1.2 pN force (see main text). A representative 2.5-pN stall (presumably from 2 dyneins) and two 1.2-pN stalls (presumably from 1 dynein) are shown for CC1-treated LPs. Counts are normalized to the maximum value for each category. For a non-normalized histogram, see Figure S2C.

See also Figure S2.

are recruited as a pair to phagosomes, resulting in peaks at ~2-pN intervals for minus directed stalls inside mammalian cells [18] and on purified phagosomes [9]. This is consistent with very recent cryoelectron microscopy (cryo-EM) investigations where two dyneins are found paired within a single DDB complex [22, 23]. Low-force peaks (below 5 pN) were not visible in our earlier measurements, likely because of abundance and/or clustering of dynein on LPs [9]. We therefore partially inactivated dynein using CC1 to measure the single-dynein force on phagosomes. Because kinesin is rarely active on LPs [9], possible effects of kinesin on dynein's force was avoided by using LPs. LPs were incubated with CC1 at a concentration where some residual motility still persisted (= 1 μM; Figure 3B). Figure 3G shows a histogram of stall forces for untreated and CC1-treated LPs [9]. In agreement with ~1.1 pN force of single dynein [9, 18, 24–28], peaks appeared at ~1.2 and ~2.3 pN for CC1-treated LPs (Figures 3G and S2C). This observation suggests that CC1 removes one dynein from some of the dynein-dynactin complexes, and the activity of these (single) remaining dyneins shows up at ~1.2 pN force. The remnant dyneins do not have impaired function because CC1-treated LPs moved with similar velocity to mock-treated LPs (1.54 ± 0.23 versus 1.39 ± 0.16 μm/s; p = 0.6).

Geometrical Considerations

We have reported an expression for the area (= $A_{CONTACT}$) from where motors on the surface of a spherical cargo may contact a MT [9]. For EPs of 750-nm diameter, $A_{CONTACT}$ is 0.08 μm^2 (≈4% of surface area $4\pi R^2$; see Figure S3A). We have shown that dynein is quite uniformly distributed on EPs by immunofluorescence [9]. Because endogenous kinesin is difficult to detect, we assume it is also uniformly distributed on EPs. With this

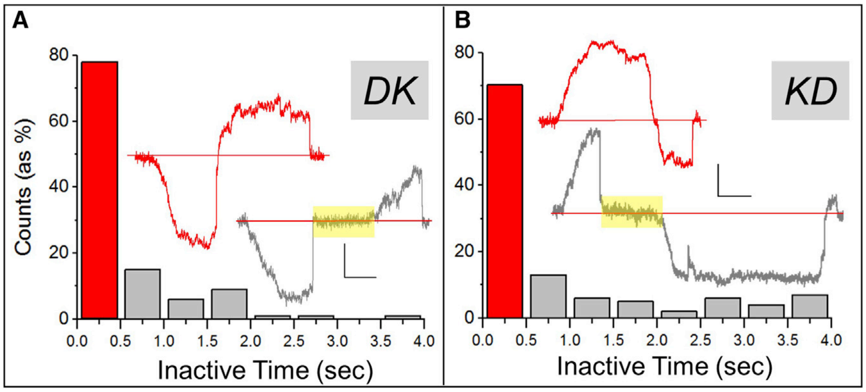

Figure 4. Distribution of Inactive Time between Stalls for EPs

(A) Histogram of inactive time observed between dynein and kinesin activity in *DK* event pairs. Reversals occurring with inactive time <0.5 s are shown in red, and those with inactive time >0.5 s are shown in gray. Insets are representative stall force records representing red and gray bins (stalls shown in corresponding colors). x scale bar, 0.5 s; y scale bar, 50 nm.

(B) Histogram of inactive time between kinesin and dynein activity in *KD* event pairs.

See also Figures S3 and S4.

assumption, ~4% of the total EP-associated kinesins or dyneins should reside within $A_{CONTACT}$. Photobleaching of vesicles (90 nm diameter) from GFP-dynactin mice shows ~3.5 dynein-dynactin subunits per vesicle [29]. Scaling by surface area, an EP of 750 nm diameter should have $Nd = 3.5 \times (750/90)^2 =$ 240 total dyneins. Because 6-fold less kinesin-1 was detected on these vesicles compared to dynein, we assume $Nk = Nd/6 = 40$. Thus, an average of $(40 \times 0.04) \approx 2$ kinesins should be present within $A_{CONTACT}$. This agrees well with peaks at ~6 and ~12 pN force on EPs corresponding to two kinesins [9]. STAR Methods discuss these issues further and presents another validation of kinesin numbers on EPs based on stall force histograms (also see Figure S3B).

Several proteins diffuse within a lipid bilayer with diffusion constant D_{LIPID} ~10 μm²/s [30]. For kinesin D_{LIPID}, ~1.4 μm²/s was reported [31]. The time spent by a diffusing protein within $A_{CONTACT}$ on an EP is $T \sim 0.08/[4 \times D_{LIPID}]$. Depending on the above values of D_{LIPID}, T ranges from 2 to 14 ms. Because reversals and stalls usually extend for ~1 s (Figures 1A and 1B), diffusion of motors (both into and out of $A_{CONTACT}$) happens much faster. Therefore, the averaged forces measured here are unaffected by lateral diffusion of motors on the phagosome membrane, if indeed such diffusion happens. In support of this, the plus-directed stall force histograms appear identical [18, 32] for lipid-enclosed phagosomes and kinesin-coated beads (where kinesin cannot diffuse around).

Kinetics of Reversals Can Be Explained by Simple Probability Calculations

Based on the above, we proceed with the assumption that ~10 dyneins and ~2 kinesins reside within $A_{CONTACT}$ and may therefore generate force to drive the EP. Because "RRs with tug-of-war" have no inactive period, one or the other class of motors is always attached to the MT. This should prevent thermal rotation of the EP during the RR. For "RRs without tug-of-war," the EP fly-back time is <5 ms (Figure 2B), this fly-back being rapidly followed by attachment of opposing motors. The EP undergoes negligible thermally driven rotation during the fly-back (<1°; estimated from rotational diffusion coefficient of a sphere). Therefore, only motors within an area $A_{CONTACT}$ can "see" the MT during a fly-back. We have earlier presented stall force histograms for EPs (see Figure 2D in [9]). The stall force values suggest competition between ≥1 kinesin and ≥6 dyneins on EPs and that ≥6 dyneins can win a tug-of-war against 1 kinesin [3]. An RR event therefore requires that (1) ≥6 dyneins and ≥1 kinesin should both be present within $A_{CONTACT}$ and (2) they should both bind to a MT simultaneously (for "RR with tug-of-war"), or in rapid succession (for "RR without tug-of-war").

For kinesin, the unloaded MT-binding rate (K_{ON}) was estimated to be ~5 s^{-1} by analyzing the density profiles of kinesin-1 along tubes pulled from a giant unilamellar vesicle [33]. If 2 kinesins are present close to the MT, then a computer simulation shows that the average time required for MT binding by ≥1 kinesin is ~0.5 s, a result that is also verified from analytical calculations (STAR Methods; Figure S4A). For dynein, it was estimated that K_{ON} ~1.6 s^{-1} by fitting data of lipid droplet transport in *Drosophila* embryos [4]. Using this value of K_{ON}, computer simulations (Figure S4B) as well as analytical calculations show that if 10 dyneins reside within $A_{CONTACT}$ then ≥6 dyneins should bind to the MT within 0.5 s. Taken together, if ~2 kinesins and ~10 dyneins exist within $A_{CONTACT}$ and are allowed to bind the MT stochastically and independently, then ≥1 kinesin and ≥6 dyneins should both generate force within a 0.5-s time window. Can this be observed in our experiments?

Figures 4A and 4B show the distribution of inactive period within *DK* and *KD* reversals. The insets show representative reversals with or without intervening inactive periods. The bin size is chosen as 0.5 s based on the above discussion. Thus, the first bin (red) contains all events where ≥6 dyneins and ≥1 kinesin bound the MT simultaneously or in rapid succession to generate force and cause reversal. Therefore, this first bin essentially depicts the expected probability that ≥6 dyneins and ≥1 kinesin are both present within $A_{CONTACT}$ (because the EP does not rotate during fly-back). This probability is strikingly high, being 70% for *KD* and 80% for *DK* reversals. Can this be explained by a simple calculation? Let us denote $P_{\geq 6D}$ as the probability of finding ≥6 dyneins, and $P_{\geq 1K}$ as probability of finding ≥1 kinesins within $A_{CONTACT}$. If we randomly throw a motor on the EP, the probability of it landing within $A_{CONTACT}$ (i.e., a "success") is 0.04 because $A_{CONTACT}$ is ~4% of the total surface area. The joint probability of finding ≥6 dyneins and ≥1 kinesins within $A_{CONTACT}$ is just the product of two binomial functions

$$[P_{\geq 6D}] \times [P_{\geq 1K}] = \left[\sum_{I=6}^{Nd} \binom{Nd}{I} \times 0.04^{I} \times 0.96^{Nd-I}\right] \times \left[\sum_{J=1}^{Nk} \binom{Nk}{J} \times 0.04^{J} \times 0.96^{Nk-J}\right]$$

$$\binom{Nd}{I} = \frac{Nd\,!}{I\,!\;(Nd - I)!} \text{ and } \binom{Nk}{J} = \frac{Nk\,!}{J!\;(Nk - J)!}, \quad \text{(Equation 1)}$$

where $P_{\geq 6D}$ and $P_{\geq 1K}$ are, respectively, the probability for ≥ 6 successes in Nd trials and ≥ 1 successes in Nk trials. Substituting Nd = 240 and Nk = 40, we find $P_{\geq 6D} \times P_{\geq 1K}$ = 0.74. This value is in excellent agreement with the 70%–80% frequency of events within first bin (Figures 4A and 4B). Therefore, the kinetics of opposing motor activity can be explained by stochastic engagement of motors, provided the EP has a certain number of total motors on its surface (Nd ~240 and Nk ~40 for 750-nm EP).

DISCUSSION

Several models have been proposed to explain bidirectional transport [1, 6, 19], including regulatory protein induced coordination [5] and mechanical activation of opposing motors [8]. However, our data do not support either of these possibilities. We provide quantitative evidence that opposing motors engage in stochastic and independent manner to cause reversals of phagosomes. We used existing estimates of dynein/kinesin numbers on cellular cargoes [4, 33], and the estimated area from where motors can engage a MT (= $A_{CONTACT}$) to calculate the probability for force generation by ≥ 6 dyneins and ≥ 1 kinesins. The calculated probability (= 74%) is in excellent agreement with experimentally measured occurrence of RRs (Figures 4A and 4B). Taken together, EPs appear to optimize dynein and kinesin numbers on their membrane such that ≥ 6 dyneins and ≥ 1 kinesin usually have access to a single MT (for a 750-nm-diameter EP). This arrangement results in intermittent force-balanced tug-of-wars and reversals in direction of motion [3]. We believe that lipids on a cargo membrane control the number and geometrical organization of motors [9, 34, 35].

We do not claim that the stochastic tug-of-war seen here is generally applicable to all cellular cargoes. Perhaps this is a special adaptation on EPs that uses force from motors to pinch receptors off the membrane of endosomes/phagosomes for recycling. The choice between dynein and kinesin on EPs was described by the tossing a fair coin, but regulatory proteins and/or lipids may upregulate one class of motors to make this coin unfair and therefore bias transport in a desired manner. Indeed, LPs move in unidirectional manner by clustering dynein into lipid micro-domains [9]. The "coin-tossing model" presented here provides a conceptual framework and predictive capability for understanding intracellular transport. It is worth asking whether a single parameter, namely, the fairness of this coin, can describe key features of polarized transport inside cells.

STAR★METHODS

Detailed methods are provided in the online version of this paper and include the following:

- KEY RESOURCES TABLE
- CONTACT FOR REAGENT AND RESOURCE SHARING
- EXPERIMENTAL MODEL AND SUBJECT DETAILS
  - Cell Culture, Antibody and Imaging
- METHOD DETAILS
  - Optical Trapping
  - Cloning and purification of proteins
  - Estimating Motor numbers on Phagosomes
  - Counting stalls in the optical trap
  - No motors engage the MT during Inactive Periods
  - Force generated by Dynein on Cellular Organelles
  - Computer simulations for binding of Motors to MT
  - Time required for binding of Motors to MT
- QUANTIFICATION AND STATISTICAL ANALYSIS

SUPPLEMENTAL INFORMATION

Supplemental Information includes four figures and can be found with this article online at https://doi.org/10.1016/j.cub.2018.03.041.

ACKNOWLEDGMENTS

R.M. acknowledges funding from a Wellcome Trust International Senior Research Fellowship (grant WT079214MA) and a senior fellowship from the Wellcome Trust – Department of Biotechnology India alliance (grant IA/S/11/2500255). P.S. acknowledges funding from the Wellcome Trust – Department of Biotechnology India alliance as an Early Career Fellowship (grant IA/E/15/1/502298). We thank R. Jha, R. Elangovan, A. Dubey, D. Pathak, S. Thakur, V. Soppina, and P. Rathaur for help with experiments and discussions.

AUTHOR CONTRIBUTIONS

R.M. wrote the manuscript with inputs from all authors. R.M., P.S., A.D., and R.P. designed experiments. P.S., A.D., Ashim Rai, and Arpan Rai did the experiments.

DECLARATION OF INTERESTS

The authors declare no competing interests.

Received: November 27, 2017
Revised: February 3, 2018
Accepted: March 19, 2018
Published: April 26, 2018

REFERENCES

1. Hancock, W.O. (2014). Bidirectional cargo transport: Moving beyond tug of war. Nat. Rev. Mol. Cell Biol. *15*, 615–628.
2. McLaughlin, R.T., Diehl, M.R., and Kolomeisky, A.B. (2016). Collective dynamics of processive cytoskeletal motors. Soft Matter *12*, 14–21.
3. Soppina, V., Rai, A.K., Ramaiya, A.J., Barak, P., and Mallik, R. (2009). Tug-of-war between dissimilar teams of microtubule motors regulates transport and fission of endosomes. Proc. Natl. Acad. Sci. USA *106*, 19381–19386.
4. Müller, M.J., Klumpp, S., and Lipowsky, R. (2008). Tug-of-war as a cooperative mechanism for bidirectional cargo transport by molecular motors. Proc. Natl. Acad. Sci. USA *105*, 4609–4614.
5. Gross, S.P., Welte, M.A., Block, S.M., and Wieschaus, E.F. (2002). Coordination of opposite-polarity microtubule motors. J. Cell Biol. *156*, 715–724.
6. Welte, M.A. (2010). Bidirectional transport: Matchmaking for motors. Curr. Biol. *20*, R410–R413.
7. Welte, M.A., Gross, S.P., Postner, M., Block, S.M., and Wieschaus, E.F. (1998). Developmental regulation of vesicle transport in Drosophila embryos: Forces and kinetics. Cell *92*, 547–557.
8. Ally, S., Larson, A.G., Barlan, K., Rice, S.E., and Gelfand, V.I. (2009). Opposite-polarity motors activate one another to trigger cargo transport in live cells. J. Cell Biol. *187*, 1071–1082.

9. Rai, A., Pathak, D., Thakur, S., Singh, S., Dubey, A.K., and Mallik, R. (2016). Dynein clusters into lipid microdomains on phagosomes to drive rapid transport toward lysosomes. Cell *164*, 722–734.
10. Barak, P., Rai, A., Dubey, A.K., Rai, P., and Mallik, R. (2014). Reconstitution of microtubule-dependent organelle transport. Methods Enzymol. *540*, 231–248.
11. Soppina, V., Rai, A., and Mallik, R. (2009). Simple non-fluorescent polarity labeling of microtubules for molecular motor assays. Biotechniques *46*, 543–549.
12. Pollock, N., de Hostos, E.L., Turck, C.W., and Vale, R.D. (1999). Reconstitution of membrane transport powered by a novel dimeric kinesin motor of the Unc104/KIF1A family purified from Dictyostelium. J. Cell Biol. *147*, 493–506.
13. Pollock, N., Koonce, M.P., de Hostos, E.L., and Vale, R.D. (1998). In vitro microtubule-based organelle transport in wild-type Dictyostelium and cells overexpressing a truncated dynein heavy chain. Cell Motil. Cytoskeleton *40*, 304–314.
14. Mallik, R., Rai, A.K., Barak, P., Rai, A., and Kunwar, A. (2013). Teamwork in microtubule motors. Trends Cell Biol. *23*, 575–582.
15. Blocker, A., Severin, F.F., Burkhardt, J.K., Bingham, J.B., Yu, H., Olivo, J.C., Schroer, T.A., Hyman, A.A., and Griffiths, G. (1997). Molecular requirements for bi-directional movement of phagosomes along microtubules. J. Cell Biol. *137*, 113–129.
16. Desjardins, M., Huber, L.A., Parton, R.G., and Griffiths, G. (1994). Biogenesis of phagolysosomes proceeds through a sequential series of interactions with the endocytic apparatus. J. Cell Biol. *124*, 677–688.
17. Harrison, R.E., and Grinstein, S. (2002). Phagocytosis and the microtubule cytoskeleton. Biochem. Cell Biol. *80*, 509–515.
18. Rai, A.K., Rai, A., Ramaiya, A.J., Jha, R., and Mallik, R. (2013). Molecular adaptations allow dynein to generate large collective forces inside cells. Cell *152*, 172–182.
19. Welte, M.A. (2004). Bidirectional transport along microtubules. Curr. Biol. *14*, R525–R537.
20. Kobayashi, T., Miyashita, T., Murayama, T., and Toyoshima, Y.Y. (2017). Dynactin has two antagonistic regulatory domains and exerts opposing effects on dynein motility. PLoS ONE *12*. Published online August 29, 2017. https://doi.org/10.1371/journal.pone.0183672.
21. Belyy, V., Schlager, M.A., Foster, H., Reimer, A.E., Carter, A.P., and Yildiz, A. (2016). The mammalian dynein-dynactin complex is a strong opponent to kinesin in a tug-of-war competition. Nat. Cell Biol. *18*, 1018–1024.
22. Urnavicius, L., Lau, C.K., Elshenawy, M.M., Morales-Rios, E., Motz, C., Yildiz, A., and Carter, A.P. (2018). Cryo-EM shows how dynactin recruits two dyneins for faster movement. Nature *554*, 202–206.
23. Grotjahn, D.A., Chowdhury, S., Xu, Y., McKenney, R.J., Schroer, T.A., and Lander, G.C. (2018). Cryo-electron tomography reveals that dynactin recruits a team of dyneins for processive motility. Nat. Struct. Mol. Biol. *25*, 203–207.
24. Mallik, R., Carter, B.C., Lex, S.A., King, S.J., and Gross, S.P. (2004). Cytoplasmic dynein functions as a gear in response to load. Nature *427*, 649–652.
25. Mallik, R., Petrov, D., Lex, S.A., King, S.J., and Gross, S.P. (2005). Building complexity: An in vitro study of cytoplasmic dynein with in vivo implications. Curr. Biol. *15*, 2075–2085.
26. McKenney, R.J., Vershinin, M., Kunwar, A., Vallee, R.B., and Gross, S.P. (2010). LIS1 and NudE induce a persistent dynein force-producing state. Cell *141*, 304–314.
27. Nicholas, M.P., Höök, P., Brenner, S., Wynne, C.L., Vallee, R.B., and Gennerich, A. (2015). Control of cytoplasmic dynein force production and processivity by its C-terminal domain. Nat. Commun. *6*, 6206.
28. Ori-McKenney, K.M., Xu, J., Gross, S.P., and Vallee, R.B. (2010). A cytoplasmic dynein tail mutation impairs motor processivity. Nat. Cell Biol. *12*, 1228–1234.
29. Hendricks, A.G., Perlson, E., Ross, J.L., Schroeder, H.W., 3rd, Tokito, M., and Holzbaur, E.L. (2010). Motor coordination via a tug-of-war mechanism drives bidirectional vesicle transport. Curr. Biol. *20*, 697–702.
30. Weiß, K., Neef, A., Van, Q., Kramer, S., Gregor, I., and Enderlein, J. (2013). Quantifying the diffusion of membrane proteins and peptides in black lipid membranes with 2-focus fluorescence correlation spectroscopy. Biophys. J. *105*, 455–462.
31. Grover, R., Fischer, J., Schwarz, F.W., Walter, W.J., Schwille, P., and Diez, S. (2016). Transport efficiency of membrane-anchored kinesin-1 motors depends on motor density and diffusivity. Proc. Natl. Acad. Sci. USA *113*, E7185–E7193.
32. Blehm, B.H., Schroer, T.A., Trybus, K.M., Chemla, Y.R., and Selvin, P.R. (2013). In vivo optical trapping indicates kinesin's stall force is reduced by dynein during intracellular transport. Proc. Natl. Acad. Sci. USA *110*, 3381–3386.
33. Leduc, C., Campàs, O., Zeldovich, K.B., Roux, A., Jolimaitre, P., Bourel-Bonnet, L., Goud, B., Joanny, J.F., Bassereau, P., and Prost, J. (2004). Cooperative extraction of membrane nanotubes by molecular motors. Proc. Natl. Acad. Sci. USA *101*, 17096–17101.
34. Steinberg, B.E., and Grinstein, S. (2008). Pathogen destruction versus intracellular survival: The role of lipids as phagosomal fate determinants. J. Clin. Invest. *118*, 2002–2011.
35. Pathak, D., and Mallik, R. (2017). Lipid-motor interactions: Soap opera or symphony? Curr. Opin. Cell Biol. *44*, 79–85.
36. Barak, P., Rai, A., Rai, P., and Mallik, R. (2013). Quantitative optical trapping on single organelles in cell extract. Nat. Methods *10*, 68–70.
37. Carter, B.C., Shubeita, G.T., and Gross, S.P. (2005). Tracking single particles: A user-friendly quantitative evaluation. Phys. Biol. *2*, 60–72.
38. Leopold, P.L., McDowall, A.W., Pfister, K.K., Bloom, G.S., and Brady, S.T. (1992). Association of kinesin with characterized membrane-bounded organelles. Cell Motil. Cytoskeleton *23*, 19–33.
39. Gross, S.P., Tuma, M.C., Deacon, S.W., Serpinskaya, A.S., Reilein, A.R., and Gelfand, V.I. (2002). Interactions and regulation of molecular motors in Xenopus melanophores. J. Cell Biol. *156*, 855–865.
40. Tabb, J.S., Molyneaux, B.J., Cohen, D.L., Kuznetsov, S.A., and Langford, G.M. (1998). Transport of ER vesicles on actin filaments in neurons by myosin V. J. Cell Sci. *111*, 3221–3234.
41. Erickson, R.P., Jia, Z., Gross, S.P., and Yu, C.C. (2011). How molecular motors are arranged on a cargo is important for vesicular transport. PLoS Comput. Biol. *7*, e1002032.
42. Nelson, S.R., Trybus, K.M., and Warshaw, D.M. (2014). Motor coupling through lipid membranes enhances transport velocities for ensembles of myosin Va. Proc. Natl. Acad. Sci. USA *111*, E3986–E3995.
43. Kunwar, A., Vershinin, M., Xu, J., and Gross, S.P. (2008). Stepping, strain gating, and an unexpected force-velocity curve for multiple-motor-based transport. Curr. Biol. *18*, 1173–1183.
44. Hendricks, A.G., Holzbaur, E.L., and Goldman, Y.E. (2012). Force measurements on cargoes in living cells reveal collective dynamics of microtubule motors. Proc. Natl. Acad. Sci. USA *109*, 18447–18452.

STAR★METHODS

KEY RESOURCES TABLE

Reagent or Resource	Source	Identifier
Antibodies		
Dictyostelium Rab7	This manuscript	N/A
Dictyostelium dynein heavy chain	This manuscript	N/A
Biological Samples		
Purified Kinesin-1	Goat brain	Described in Ref [36]
Chemicals, Peptides, and Recombinant Proteins		
HL5 media	Formedium	HLG0102
Penicillin/Streptomycin	GIBCO	15140122
750nm carboxylate polystyrene beads	Polysciences	07759-15
Phenyl methane sulfonyl fluoride (PMSF)	Sigma	T8830
5 micron syringe filter	Sartorius	16517E
ATP	Sigma	34369-07-8
Benzamidine	Sigma	1670-14-0
Lysozyme	Sigma	62971
Glutathione Sepharose ™ 4B	Amersham	27- 4574-01
Thrombin	Sigma	9002-04-4
IPTG	Sigma	367-93-1
Tris	M. P. Biomedicals	819623
NaCl	Merck	7647-14-5
DTT	Sigma	3483-12-3
$CaCl_2$	Sigma	449709
Experimental Models: Cell Lines		
Dictyostelium discoideum (AX2)	Rob Kay lab	Axenic strain (DBS0235521)

CONTACT FOR REAGENT AND RESOURCE SHARING

Further information and requests for resources and reagents should be directed to and will be fulfilled by the Lead Contact, Roop Mallik (roop@tifr.res.in).

EXPERIMENTAL MODEL AND SUBJECT DETAILS

Cell Culture, Antibody and Imaging

Dictyostelium discoideum cells were cultured axenically in HL5 medium (ForMedium) containing 100μg/ml penicillin-streptomycin. Antibody against *Dictyostelium* Rab7 was generated in rabbit against a peptide. Antibody against *Dictyostelium* dynein heavy chain was generated in mice using the purified dynein stalk head domain as an antigen. Latex bead phagosomes were prepared as previously described [9, 36]. Briefly, *Dictyostelium* cells were incubated with 750nm polystyrene beads and chased (5 min for EPs; 40 min for LPs) at 22°C. The cells were then pelleted at 4°C, washed and lysed using a 5μm pore-sized syringe filter. The lysate was centrifuged, phagosomes were collected along with the high-speed supernatant and frozen in liquid nitrogen. Polarity marked microtubules were prepared and motility of phagosomes was assayed at 1mM ATP concentration [11]. The assay has been described previously and used to observe motion of endosomes and phagosomes in cell extracts and inside cells [9, 18, 36]. Phagosome motion was observed using differential interference contrast microscopy (DIC) with a 100X, 1.4 NA oil objective (Nikon). Image acquisition was done at video rates (30 frames/sec no binning) with a Cohu 4910 camera. Position tracking of phagosomes was done with custom written software in Labview (National Instruments) using an algorithm which calculates position of the centroid of a cross-correlation image with sub-pixel resolution [37].

METHOD DETAILS

Optical Trapping

The setup has been described [36]. A single mode diode laser at 980nm was used. The laser power at the sample plane was between 20-70 mW. A quadrant photodiode (QPD) was used to obtain stall force records and thermal fluctuation data for measuring trap stiffness. Stall force data was digitized at 2 kHz. Thermal fluctuations were recorded at 40 kHz. For measuring trap stiffness of phagosomes, the video-matching method (V_{MATCH}) was used. The linear range of both the QPD and optical trap had been measured separately. The chosen cut-off for measuring stalls is within the measured linear range of QPD. Stall force records were analyzed with custom written programs developed in Labview (National Instruments).

Cloning and purification of proteins

Dictyostelium p150/DynA protein sequence was aligned with rat, mouse and human p150 sequences and various domains were mapped using bioinformatics software MEME from National Biomedical Computation Resource. The overall domain structure of DynA is conserved with that of mammals. CC1 domain of DynA (implicated in binding dynein intermediate chain) was cloned into pGEX4T3 vector. GST-CC1 was then purified from bacterial cells BL21 pLys by inducing 1 l culture at 25°C for 5 hr using 1 mM IPTG. Cells were pelleted and processed for purification. Cell pellet was lysed using lysis buffer (20mM Tris, 150mM NaCl, 1mM DTT pH 7.4 with 1% Triton) complemented with protease inhibitors - 1X PIC (Roche) and 1mM PMSF (Sigma T8830). Lysate was incubated with 1mg/ml lysozyme (Sigma 62971) at 4°C for 30 mins and further sonicated 5 times for 10 s with 30 s gaps on ice. After lysis, the debris and membrane were pelleted. The clarified lysate was then incubated with 1 mL GST-Beads (Glutathione Sepharose™ 4B from Amersham Biosciences 27-4574-01) for 3 hr and shaken at 4°C. The flow-through was discarded and beads were washed with 10 column volumes of wash buffer (20mM Tris, 300mM Nacl, 1mM DTT pH 7.4). To remove the GST tag, washed beads were incubated with 40 units of thrombin (Sigma 9002-04-4) in 1X PBS and 1mM $CaCl_2$ at 4°C overnight. Finally, soluble CC1 was separated from the GST-bound beads by a low speed spin. To inactivate thrombin 5mM Benzamidine (Sigma 434760) was added to protein solution and protein was stored in aliquots at −80 degrees. For CC1 treatment, 0.4-0.45 μg of CC1 protein in PBS was added to one aliquot of EPs, incubated on ice for 10 mins followed by motility assay. For mock treatment, instead of protein, same volume of 1X PBS was added to EP aliquot. For our experiments, each aliquot of EP (36 μl) contains 5×10^7 to 10^8 phagosomes mixed in cytosol. Kinesin-1 was purified from goat brains using a nucleotide dependent microtubule affinity procedure and bead assays were performed as described [36].

Estimating Motor numbers on Phagosomes

Immuno-EM studies have shown presence of about 200 kinesins per mitochondria [38]. A single melanosome (~500nm diameter) in *Xenopus* melanophores is estimated to have ~88 myosin-V motors on it by quantitative western blotting [39]. This estimate is in good agreement with immuno-EM data showing ~120 myosins on vesicles of ~600nm diameter [40]. Therefore a cargo of 750nm diameter (same as EP) should have $88 \times (750/500)^2 = 200$ myosin motors. Erickson *et. al.* have simulated the binding to MT of motors present on a spherical cargo [41]. When a total of 30 motors is present on a cargo of diameter 250nm, it is seen that ~6 motors can bind a MT. These numbers again suggest that an EP could have $30 \times (750/250)^2 = 270$ motors on it. In a recent report, upto 15 myosins were coupled to 200nm diameter liposomes to study how motor-ensembles drive vesicle transport [42]. This suggests $15 \times (750/200)^2 = 211$ myosins on a 750nm diameter vesicle. Therefore, we believe that $Nd = 240$ is a reasonable estimate of the total number of dyneins on an EP. Of these 240 dyneins, a small fraction ($= 240 \times 0.04 = 10$ dyneins) will be active because they reside within $A_{CONTACT}$ ($= 4\%$ of total surface), and can contact a MT at one time to generate force. This conjecture is in agreement with minus-directed forces upto ~12pN seen frequently on phagosomes, presumably arising from ~10 dyneins that appear to cooperate and generate additive forces in a team [9, 18].

Considering some uncertainty/variability in the absolute number of motors on a cargo, we decided to test our results on the basis of another parameter. This parameter is the ratio of motors above and below a certain threshold. Figure S3B presents the stall force histogram for kinesin on EPs. The histogram has been fitted to a sum of two independent Gaussian distributions. This fitting reveals that ~40% force generation events correspond to a single kinesin, and ~60% events require more than a single kinesin. Assuming $A_{CONTACT} = 4\%$ and $Nk = 40$, we find from the binomial distribution that probability of finding 1 kinesin in $A_{CONTACT}$ (P_{1K}) = 32% and more than 1 kinesin ($P >_{1K}$) = 48%. The remaining 20% correspond to no kinesin being found in $A_{CONTACT}$. The use of a binomial distribution in these calculations will be elaborated in the next section. These probabilities are in reasonable agreement with the 40-60 ratio of force generation events for kinesin seen in Figure S3B. There may be some error in this estimation because unlike dynein, kinesin motors do not work cooperatively and their stall forces are not exactly additive [43].

Counting stalls in the optical trap

As discussed in the main manuscript, we will use the single-motor force values for kinesin and dynein as ~6pN and ~1.1pN respectively.

Plus-directed kinesin (K) stalls

To count an event as a *K* stall (see Figure 1A), we required that maximum Force > 3pN and $T_{STALL} > 0.5$ s. Some low-force events did not satisfy both of these criteria. They likely correspond to premature detachment of kinesin and were not counted as separate stalls [25]. For example, see the two events marked by red stars in Figure 1A (Force < 3pN and $T_{STALL} < 0.5$ s). To verify if these

are indeed premature detachments, we measured velocity of the EP in a time-window of 0.2 s (= 400 data points) just prior to detachment of motors from MT. For stalls in the premature detachment category (22 stalls used), this velocity was significantly higher than the normal stalls (Figure S1A). Therefore, stalls such as those marked with red stars in Figure 1A were counted as one *KK* event-pair and not as two *KK* event-pairs.

Minus-directed dynein (D) stalls

Unlike the plus directed stalls, low-force stalls were rarely seen for minus direction on EPs (see Figure 1A in main manuscript and Figure 2A in Ref [9].). We believe this is because of high dynein density on the EP, because dyneins appear to work as a team such that their forces and T_{STALL} add up, and because dynein also has a catch-bond to resist detachment from the MT [9, 18]. We double-checked for low-force stalls after reducing the laser power, but such events were still rare. However, reducing dynein activity on EPs by CC1 addition did result in low-force minus directed stalls (Figures 4G and S2C). This supports our view that single dynein generates ~1.1pN force on the EP, but high dynein surface density and cooperativity results in robust high-force minus directed stalls on untreated EPs.

No motors engage the MT during Inactive Periods

We trapped EPs and held them close to the coverslip, but away from MTs to measure the fluctuations in position along X and Y directions. In separate experiments we also measured the fluctuation in position of EPs perpendicular to MT (i.e., Y direction) during inactive periods at trap center between two stalls (yellow boxes in Figure 1A). The SD in perpendicular position (i.e., the fluctuation) for both cases is similar (Figure S1B), suggesting that all motors have detached during the inactive period (yellow boxes in Figure 1A). The fluctuations perpendicular to MT have minimal contribution from motion along MT driven by motors. This strategy of using perpendicular fluctuations has been used earlier [25].

Force generated by Dynein on Cellular Organelles

The force on beads coated with recombinant dynein, dynein+dynactin (DD) and dynein+dynactin+BicD (DDB) complexes has been reported recently [21]. Dynein exhibited a stall force of 2.04 ± 0.02pN, which is significantly higher than the force of ~1 pN for non-yeast dynein reported by several groups (9, 18, 24–28). The reason behind this discrepancy is unclear. DDB complexes generated 4.30 ± 0.12pN force, suggesting that single dynein within DDB complexes generates 4.3pN force [21]. In contrast to these results, our force measurement on phagosomes inside cells [18] and in reconstituted assays [3, 9] have been consistent with ~1pN force for dynein, and two dyneins functioning as a pair on cellular vesicles. This is in agreement with the low force for dynein observed by others inside cells [32, 44]. The difference in force production between DDB complexes [21] and native vesicles [3, 9, 18, 32, 44] may indicate modulation of dynein force *in vivo* by co-factors that are missing in the reconstituted DDB complexes. We hope these issues can be clarified in future through concerted progress on both fronts (bead assays and assays with native-like organelles).

Computer simulations for binding of Motors to MT

Time to MT-binding of motors is obtained from computer simulations (Figure S4). In these Monte Carlo (MC) simulations we are computing the mean first passage time for binding of 6 dynein motors to the MT based on K_{ON} and K_{OFF}. We first place an upper limit of 10 dyneins that can possibly bind to the MT (this is the number of dyneins present within $A_{CONTACT}$; see main text). We then start MC with zero dyneins bound, and all the 10 dyneins are allowed to attach/detach. This is repeated many times. Similarly, a MC simulation was done for kinesin.

Time required for binding of Motors to MT

In a simplest model, let us assume that motors bind and unbind to the MT with respective rates K_{ON} and K_{OFF}. The mean density of bound motors *C(t)* will then obey the equation

$$\frac{dC}{dt} = K_{ON}(1 - C) - K_{OFF}C \,. \tag{1}$$

This has a solution

$$C(t) = C_{eq}[1 - \exp(-(K_{ON} + K_{OFF})t)] \,, \tag{2}$$

where C_{eq} is the equilibrium density of motors, which can be obtained from Eq. 1 by equating the left hand side to zero. By doing so, we obtain

$$C_{eq} = \frac{K_{ON}}{K_{ON} + K_{OFF}} \tag{3}$$

from Eq. 2, when $t = \frac{1}{K_{ON} + K_{OFF}}$ we have

$$C\,(t) = C_{eq}\left(1 - \frac{1}{e}\right) = C_{eq} \times 0.63 \,. \tag{4}$$

The characteristic time it takes to reach a mean density of MT-bound motors (= 63% of C_{eq}) will be

$$t = \frac{1}{K_{ON} + K_{OFF}}. \quad (5)$$

Now, if total dyneins on EP = Nd = 240, then dyneins within $A_{CONTACT}$ = 240 × 0.04 ~10. For dynein we use $K_{ON} =$ 1.6 s^{-1} and K_{OFF} = 0.27 s^{-1} [4]. Therefore, C_{eq} = 0.85. The mean density of bound motors is then (10 × 0.85 × 0.63 = 5.5). So, ~6 dyneins would bind to the MT in t = 0.53 s (obtained from Eq. 5 after substituting values of K_{ON} and K_{OFF}).

Similarly, if total kinesins on EP = Nk = 40, then kinesins within $A_{CONTACT}$ = 40 × 0.04 ~2. For kinesin we use $K_{ON} =$ 5 s^{-1} and K_{OFF} = 1 s^{-1} [4]. Therefore, C_{eq} = 0.83. The mean density of bound motors is then (2 × 0.83 × 0.63 = 1). So, 1 kinesin would bind to the MT in t = 0.16 s.

Therefore, if ~2 kinesins and ~10 dyneins exist within $A_{CONTACT}$, and they bind the MT independent of each other, then ≥ 1 kinesin and ≥ 6 dyneins will ***both*** bind the MT and generate force within ~0.5 s.

QUANTIFICATION AND STATISTICAL ANALYSIS

The OriginLab package was used for data representation and statistical analysis. Student's t test (unpaired, two tailed) was used to calculate statistical significance for difference between means (95% confidence).

Current Biology, Volume *28*

Supplemental Information

Coin Tossing Explains the Activity of Opposing Microtubule Motors on Phagosomes

Paulomi Sanghavi, Ashwin D'Souza, Ashim Rai, Arpan Rai, Ranjith Padinhatheeri, and Roop Mallik

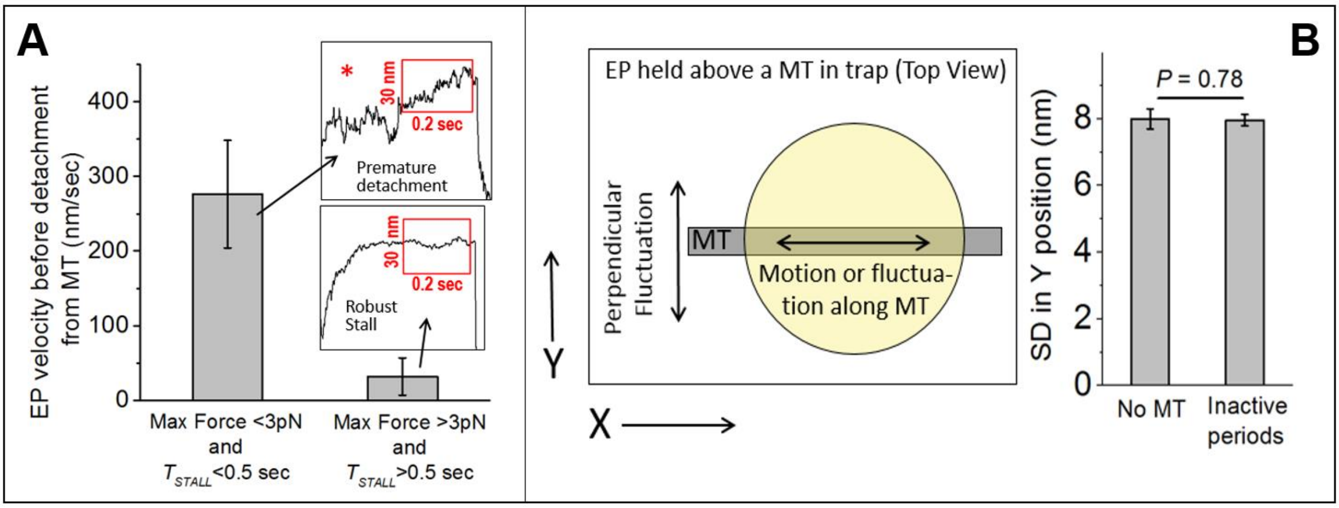

Figure S1. Scoring stalls in the trap and absence of motor activity during inactive periods, related to Figure 1 and Figure 2

A) Identification of premature detachment of Kinesin in an optical trap. Plus directed stalls were divided into two categories based on the criterion mentioned on X-axis of this Figure. The velocity in a 200ms window prior to detachment was measured by linear fitting. The insets show examples of each category and the boxed regions used to calculate velocity. Significantly higher velocity was seen in the first category (red star), suggesting that these are premature stalls.

B) Left Panel:- The SD in position (i.e. the fluctuation) of EPs along Y direction in an optical trap, shown as a schematic. The SD along Y (and not X) was chosen to minimize effect of motor-driven excursions along X.

Right panel:- **"No MT"**-- EPs were caught in the optical trap and held above the coverslip in absence of MTs. **"Inactive periods"** -- examples are shown as yellow boxes in Figure 1A. EPs resided at the centre of the optical trap and did not show significant motion along the MT (i.e. in X direction) during Inactive periods. The SD in position along Y direction during "Inactive period" is plotted. This is equal to the SD for EPs that had no MT underneath them. This suggests that EP-associated motors do not attach to the MT during inactive periods.

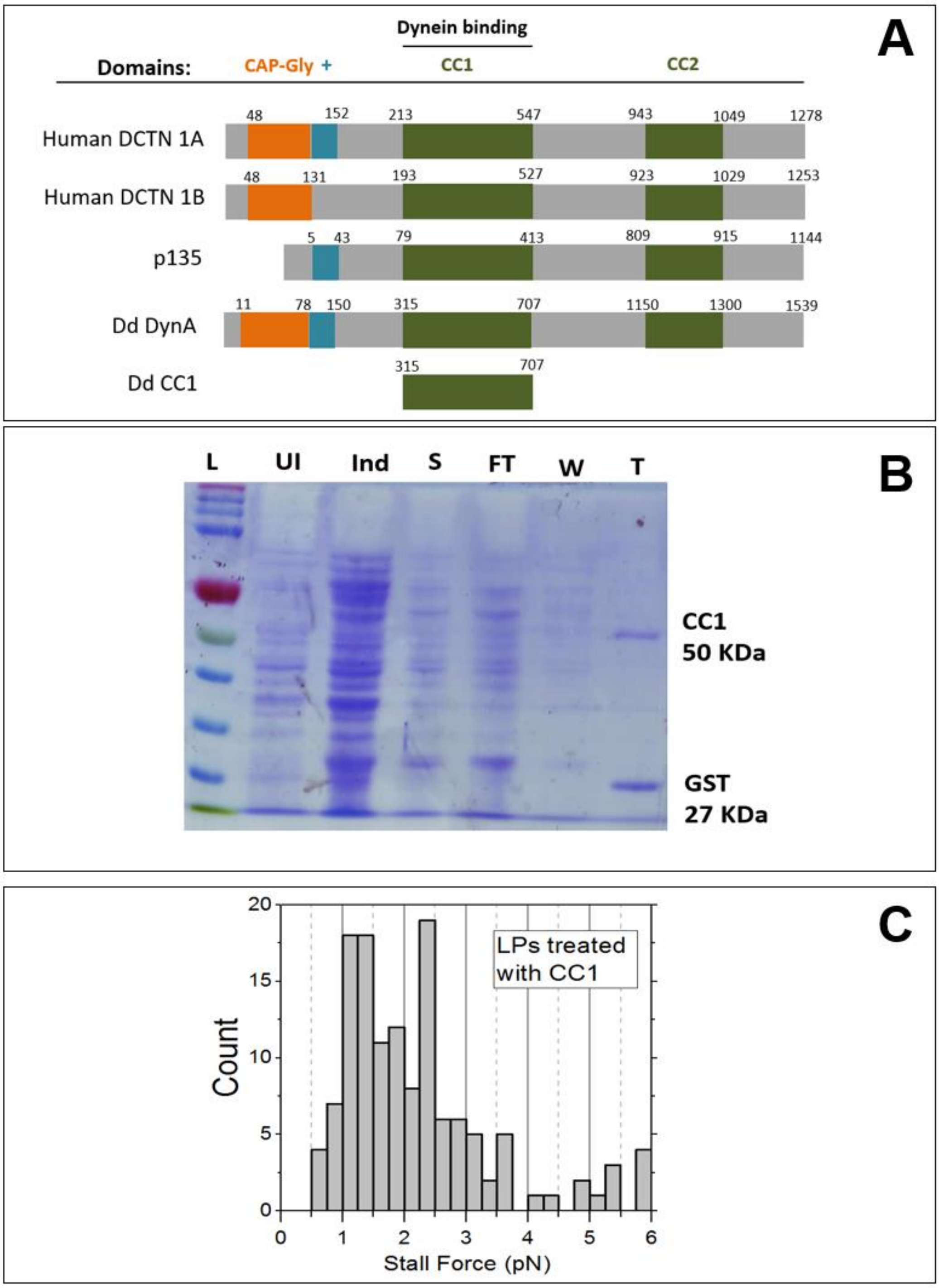

Figure S2. CC1 domains, purification and force measurement of LPs, related to Figure 3

A) Schematic representations of dynactin/p150 isoforms from Human and *Dictyostelium discoideum* (Dd). The CAP–Gly, basic (+), coiled-coil 1 and 2 (CC1 and CC2) domains for each isoform are labeled. The cloned fragment used in motility inhibition assays (Dd CC1) is shown. Numbers indicated on each isoform refer to their corresponding amino-acids.

B) GST-CC1 was purified from BL21 pLys cells using GST sepharose beads. Following are the fractions loaded on the gel. L: Protein Ladder, UI: Uninduced sample, Ind: Induced sample (1mM IPTG, 25 degrees C, 5 hrs), S: Soluble fraction post lysis and spin FT: Flow through W: Wash T : Fraction collected after thrombin cleavage.

C) Stall force histogram for LPs treated with 1 micromolar CC1. The bin size is 0.25pN. Very few counts are seen above 4pN force. The lowest peak is seen at ~1.2pN, suggesting that this is the force exerted by single dynein on CC1 treated LPs. The peak at ~2.2pN is suggestive of force from a pair of dynein motors (see main text).

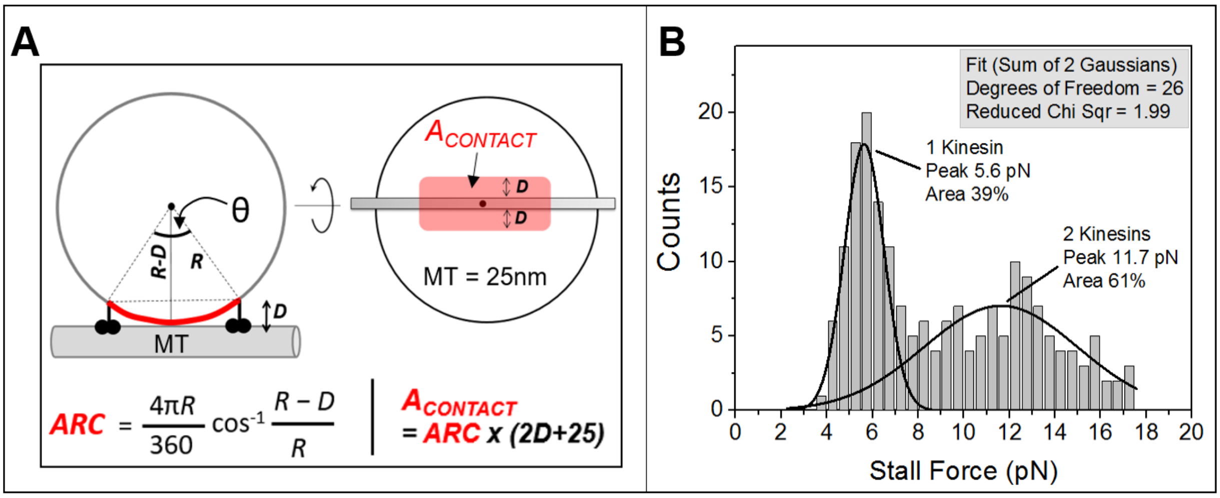

Figure S3. Geometry of phagosome-MT contact and Kinesin stall force histogram, related to Figure 1 and Figure 4

A) Left panel: A spherical cargo of radius R contacts the MT with motors of length *D*. The red line represents an arc from where motors can bind the MT. Motors associated with the cargo outside this region are not long enough to bind the MT. The length of this arc is shown, and the angle subtended by it at the cargo centre (θ) is also shown. **Right panel:** The cargo is rotated as shown. Motors present along the arc on either side of the MT can engage if the perpendicular distance between the point of attachment on the cargo and the MT is less than the length of the motor. The area $A_{CONTACT}$ on the cargo from where motors can contact the MT is shown in red. This area is a rectangle with length equal to the length of the arc and breadth equal to *D+25+D*. For 750nm dia cargoes, $A_{CONTACT}$ is ~4% of the total surface area.

B) Histogram of stall forces of kinesin measured on EPs purified from *Dictyostelium*. The distribution can be fitted to two individual Gaussians: one with a peak at 5.6pN corresponding to 1 kinesin events and other with a 2 kinesin peak at 11.7pN. The gray box reports the fit parameters.

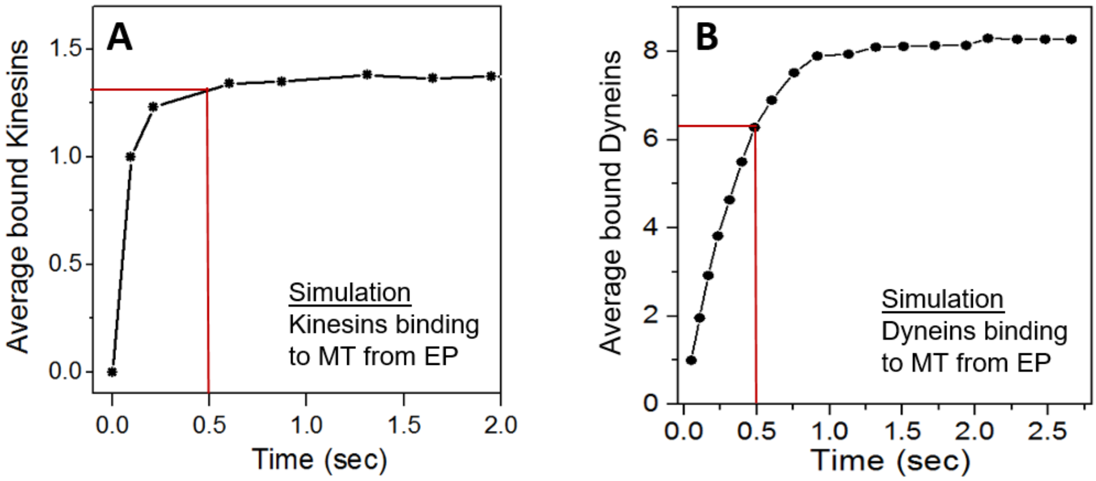

Figure S4, related to Figure 4

Average number of MT-bound motors (simulation).

A) Average number of bound kinesins as a function of time obtained from simulations. 2 kinesins are placed close to a MT (i.e. within $A_{CONTACT}$) and allowed to bind/unbind from the MT (see main text for more details).

B) Average number of dyneins bound to MT as a function of time obtained from simulations. 10 dyneins are placed close to a MT (i.e. within $A_{CONTACT}$) and allowed to bind/unbind from the MT (see main text for more details).
